# Desmosterol-driven atypical macrophage polarization regulates podocyte dynamics in diabetic nephropathy

**DOI:** 10.1007/s11033-023-09198-3

**Published:** 2024-01-27

**Authors:** Huiying Qi

**Affiliations:** Department of Cardiology, Branch of Tianjin Third Central Hospital, 220 Jiangdu Road, Tianjin, 300250 China

**Keywords:** Diabetic nephropathy, Macrophages, Polarization, Podocytes, Desmosterol

## Abstract

**Background:**

Diabetic nephropathy (DN) stands as a leading diabetes complication, with macrophages intricately involved in its evolution. While glucose metabolism’s impact on macrophage activity is well-established, cholesterol metabolism’s contributions remain less explored. Our study seeks to elucidate this association.

**Methods and results:**

Methods and Results: Gene expression analysis of monocytes from the blood of both normal and diabetic patients was conducted using public databases, showing that cholesterol metabolism pathways, especially Bloch and Kandutsch-Russell, were more altered in diabetic monocytes/macrophages than glucose-responsive pathways. When bone marrow-derived macrophages (BMDMs) were subjected to desmosterol, they exhibited an unconventional polarization. These BMDMs displayed heightened levels of both M1-related pro-inflammatory cytokines and M2-linked anti-inflammatory factors. Further, in co-culture, desmosterol-conditioned BMDMs paralleled M2 macrophages in augmenting Ki-67 + podocyte populations while mimicking M1 macrophages in elevating TUNEL + apoptotic podocytes. Comparable outcomes on podocytes were obtained using conditioned media from the respective BMDMs.

**Conclusions:**

Our data underscores the pivotal role of cholesterol metabolism, particularly via desmosterol, in steering macrophages toward an unconventional polarization marked by both inflammatory and regulatory traits. Such unique macrophage behavior concurrently impacts podocyte proliferation and apoptosis, shedding fresh light on DN pathogenesis and hinting at potential therapeutic interventions.

## Introduction

Diabetes is a widespread metabolic disorder impacting countless individuals globally. Those with diabetes grapple with the inability to regulate blood glucose levels due to diminished insulin production, reduced cellular sensitivity to insulin, or a combination of both [1]. Diabetic nephropathy (DN) is one of the leading complications of diabetes, characterized by renal dysfunction that can progress to end-stage renal disease [2]. The intricate interplay between metabolic disturbances and immune responses underpins the pathogenesis of DN. Among the diverse immune cells implicated in DN, macrophages stand out due to their remarkable phenotypic plasticity and pivotal role in inflammation and tissue remodeling [3].

Traditionally, macrophages are categorized into two main polarization states: M1 (pro-inflammatory) and M2 (anti-inflammatory or reparative) [4]. M1 macrophages are typically induced by stimuli such as lipopolysaccharide (LPS) and interferon-gamma (IFN-γ) and are known for their role in mediating acute inflammatory responses, whereas M2 macrophages, induced by factors like interleukin-4 (IL-4) and interleukin-13 (IL-13), are associated with tissue repair and the resolution of inflammation [5]. However, the binary M1/M2 classification is an oversimplification, as macrophages can adopt a spectrum of activation states influenced by a variety of environmental cues [6]. In the context of DN, the polarization state of macrophages can influence key renal cell types, notably podocytes, which are integral to maintaining the glomerular filtration barrier [7]. Disturbances in podocyte function, including altered proliferation and increased apoptosis, are hallmarks of DN [8–12].

While the impact of glucose metabolism on macrophage function in DN has been extensively studied, recent evidence suggests a paramount role for cholesterol metabolism, particularly through the Bloch and Kandutsch-Russell pathways [13]. Cholesterol, a vital component of cell membranes, also serves as a precursor for several bioactive molecules. Its biosynthesis, involving intermediates such as desmosterol, appears to be more than just a metabolic pathway; it seems to be intricately linked to immune modulation [14]. Desmosterol, an intermediate in cholesterol synthesis [15], has been highlighted of its potential role as an endogenous modulator of macrophage function [16] and of its elevation during diabetes [17]. However, the connection between cholesterol metabolism, especially the role of desmosterol, and macrophage polarization in the context of DN, is largely unknown. In this study, by delving into how desmosterol influences macrophage phenotype and its subsequent effects on podocyte dynamics, we aim to provide fresh insights into the pathogenesis of DN.

## Materials and methods

### Experimental protocols and reagents

The Tianjin Third Central Hospital’s Animal Research and Care Committee sanctioned all experimental procedures. Male C57BL/6 mice, 12 weeks old and with a weight range of 25 to 30 g, were acquired from the Shanghai Laboratory Animal Research Center, China.

### Mouse BMDM preparation and differentiation procedure

The subsequent procedure delineates the extraction, cultivation, and differentiation steps of mouse bone marrow-derived macrophages (BMDMs) into either M1 or M2 macrophages. Initially, ensure 12-week-old male C57/Bl6 mice are euthanized adhering to recommended guidelines. Sterilize the rear limbs using a 70% ethanol solution before excising the femurs and tibias. Any residual muscle tissue should be meticulously removed. Subsequently, place the bone fragments in a sterile Petri dish with phosphate-buffered saline (PBS; Sigma-Aldrich, Shanghai, China).

With sterile precision tools, snip the bone extremities, then employ a syringe, pre-filled with cold PBS (with an added 2% of fetal bovine serum (FBS)), to extract the bone marrow. This extraction should be gathered in a sterile 50mL container. Disperse any aggregated cells by gently agitating the bone marrow solution. Following this, to sift out any remnants and achieve a singular cell consistency, sieve the fluid through a 70 μm cell filter.

The next phase involves centrifugation of the cell mix at a force of 300 x g for a duration of 5 min. The supernatant post-centrifugation is redundant and should be discarded. The residual cell collection should then be suspended in DMEM (Sigma-Aldrich) enhanced with 10% FBS, a 1% blend of penicillin-streptomycin, and 25ng/mL of macrophage colony-stimulating factor (M-CSF; Sigma-Aldrich). Quantify the cells using a hemocytometer and distribute them at a concentration of 10^6^ cells within each 10 cm dish or a suitable cell cultivation container.

Position the cells in a controlled environment of 37 °C with 5% CO2 humidity for a span of 6 days, facilitating the transformation of marrow cells into macrophages. Every third day, replenish the medium containing M-CSF. Post-differentiation, gather the macrophages with a cell scraper and transfer them to fresh culture dishes according to the needed density.

For the final differentiation into M1 or M2 states, the macrophages should be exposed to distinct agents. The M1 state requires the addition of 100 ng/mL lipopolysaccharide (LPS; Sigma-Aldrich) combined with 20ng/mL of IFNγ (also from Sigma-Aldrich), followed by a 48-hour incubation. Conversely, achieving the M2 state necessitates the inclusion of 20ng/mL Il4 and Il13 (Sigma-Aldrich) to the medium, again with a subsequent 48-hour incubation. For desmosterol treatment (D6513, Sigma-Aldrich), 10 µg/mL was used.

### Mouse podocyte isolation

Euthanize the mouse and expose the abdominal cavity. Carefully extract the kidneys, ensuring minimal damage to the tissue. Once extracted, place the kidneys in PBS to wash away any extraneous blood and debris. Subsequently, decapsulate the kidneys by gently removing the outer layer with fine-tipped forceps. After decapsulation, mince the kidneys finely using sterile scalpels. Place these minced tissues in a digestion solution containing a mix of 0.1% collagenase type II and 5 mg/mL DNase I to facilitate disaggregation of cells. Incubate the mixture at 37 °C with gentle agitation for about 30 min until the tissue is fully digested. Following digestion, pass the cell suspension through a 40 μm cell strainer to separate podocytes and other kidney cells from undigested tissue fragments. The filtrate is then centrifuged at 300 x g for 5 min to pellet the cells. Discard the supernatant and resuspend the pellet in podocyte-specific culture medium (Dulbecco’s Modified Eagle Medium (DMEM) supplemented with 10% FBS, 1% Insulin-Transferrin-Selenium (ITS), 15mM HEPES Buffer, 2mM L-Glutamine and 1% 100 U/mL penicillin and 100 µg/mL streptomycin). To further purify podocytes, take advantage of their adherence properties: plate the cell mixture onto culture dishes and incubate for a limited period (usually 1–2 h) at 37 °C. Non-adherent cells are then removed by gentle washing with warm PBS, leaving a relatively pure population of adherent podocytes. These can be maintained in a humidified incubator at 37 °C with 5% CO_2_, ensuring the medium is refreshed every 2–3 days. It’s critical to note that primary podocytes undergo limited replication in culture, so timely experiments or sub-culturing is advised.

### Co-culture

The transwell co-culture system (8 μm pore size, Corning Co., NY, USA) is an advanced method employing semi-permeable membrane inserts that are strategically positioned within cell culture wells, bathed in their corresponding media. For each chamber, a concentration of 200 µl containing 105 cells/ml was meticulously maintained. A total of 96 h was used to co-culture the cells. To evaluate the interaction between podocytes and macrophages, a microfluidic chamber system was employed. Quantification of the cell population was adeptly carried out using the CCK-8 assay, sourced from ThermoFisher Scientific, Beijing, China. Desmosterol was used at a final concentration of 10µM.

### ELISA

RIPA buffer was used to lyse cells to collect proteins. ELISA kits for mouse IL-1β (Ab197742, Abcam), mouse tumor necrosis factor alpha (TNFα; Ab208348, Abcam), mouse interferon gamma (IFNɣ; Ab282874, Abcam), mouse CD163 (Ab272204, Abcam), mouse CD206 (LS-F17964, LSBio, Shanghai, China), mouse IL-10 (M1000B; R&D System), mouse vascular endothelial growth factor A (VEGF-A, BMS619-2, Invitrogen, Beijing, China) and mouse transforming growth factor beta 1 (TGFβ1, DY1679, R&D System) were utilized as per the manufacturer’s instructions.

### Ki-67 and TUNEL assays

The Ki-67 assay was performed using a specific antibody sourced from Thermo Fisher Scientific. The TUNEL assay was conducted utilizing a dedicated detection kit provided by Roche Applied Science (Nutley, NJ, USA). The percentages of proliferating and apoptotic cells were determined by dividing the number of Ki-67 + or TUNEL + cells by the total cell count, respectively.

### Bioinformatics and statistical analyses

We delved into established databases focusing on monocytes/macrophages in human DN research, singling out the GSE65561 database [18]. This specific database provides RNAseq results from monocytes drawn from both diabetic and healthy subjects. We utilized the GEO2R online bioinformatics platform, incorporating R programming, to extract individual data points. To ensure data integrity, we employed a boxplot to showcase gene expression data spread, and a UMAP plot to visualize variance distribution between the groups under study. Additionally, a mean-difference plot illustrated average gene expression disparities between the two groups, based on a set statistical benchmark. For a detailed analysis, we crafted a visual representation pinpointing genes that notably deviated in expression in the diabetic cohort compared to the control, considering both statistical significance (*p* < 0.05) and a substantial fold change (LogFC > 0.5 or LogFC<-0.5). Our findings in this study are articulated through individual data points, supplemented by the mean and standard deviation (SD). For the statistical scrutiny, we employed a one-way ANOVA, complemented by Bonferroni post-hoc adjustments for comprehensive comparison checks (GraphPad Prism, GraphPad Software, Inc., La Jolla, CA, USA). Results with a *p*-value below 0.05 were deemed significant, whereas “NS” indicated a lack of significant disparities between groups. We also integrated Pearson’s correlation coefficient to gauge the robustness and trajectory of relationships between our study variables.

## Results

### Human study highlights the significance of cholesterol metabolism responses in diabetic monocytes/macrophages

To elucidate the molecular mechanisms by which macrophages influence the onset of DN, we initially sought to identify primary alterations in monocytes/macrophages due to diabetes. To achieve this, we conducted gene expression analyses on monocytes sourced from the blood of both healthy and diabetic individuals using public databases. Our exploration of published databases led us to a dataset (GSE65561) that offered RNAseq outcomes on monocytes from these two patient groups.

**Fig. 1:**
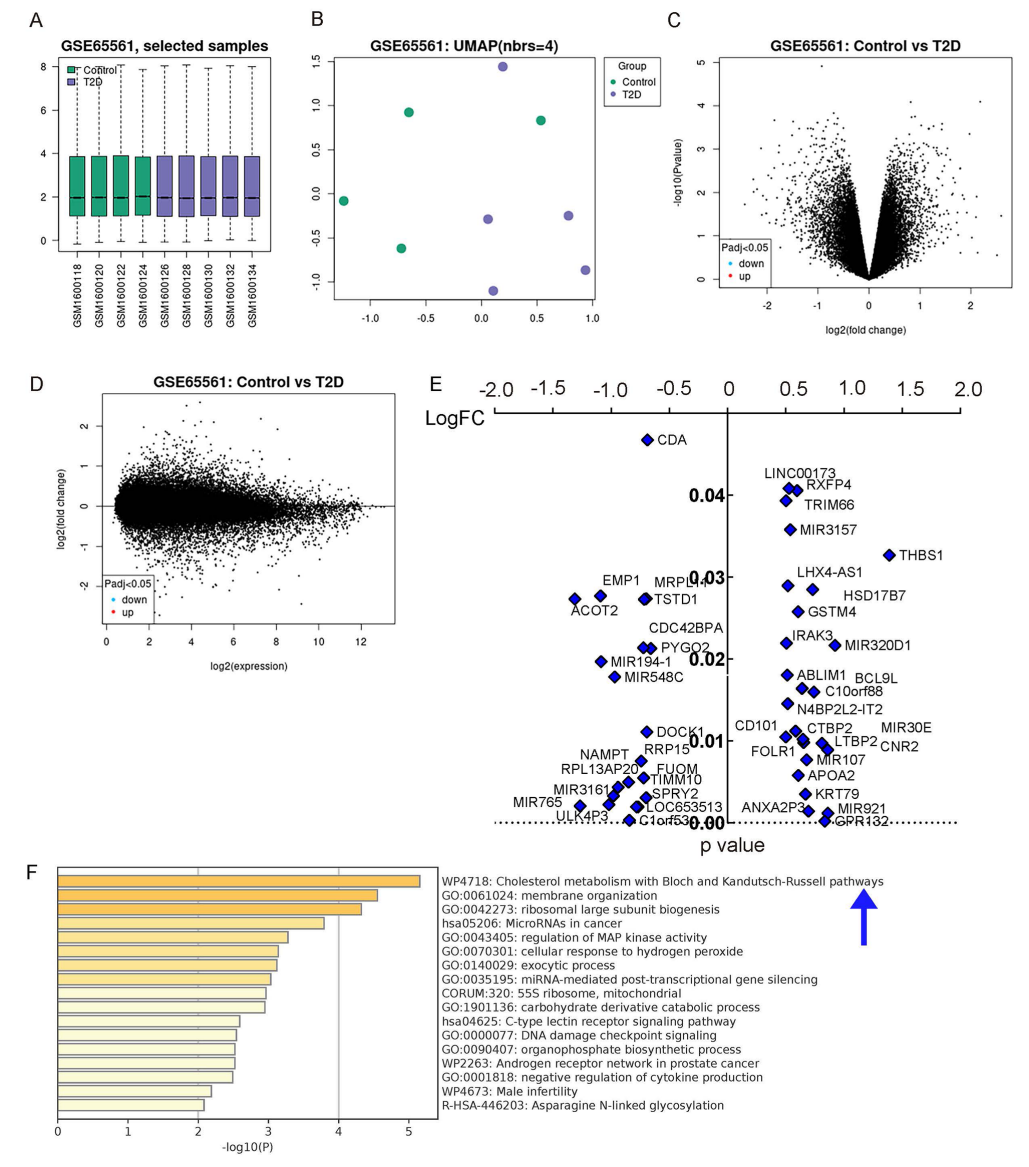
Human study highlights the significance of cholesterol metabolism responses in diabetic monocytes/macrophages. Gene expression analyses on monocytes sourced from the blood of both healthy and diabetic individuals using public database GSE65561. **(A)** A gene expression distribution boxplot. **(B)** A UMAP plot assessing variable distribution across the studied groups. **(C-D)** Mean-difference plots illustrating the average gene expression levels between normoglycemic controls (control) and type 2 diabetes (T2D) subjects. **(E)** A figure to spotlight genes that were significantly (*p* < 0.05) and substantially (LogFC > 0.5 or LogFC<-0.5) upregulated or downregulated in the T2D group relative to the control group. **(F)** Pathway analysis linked with these markedly altered genes in diabetic monocytes with the online tools available at Metascape.com

We employed the GEO2R online bioinformatics tool combined with R language scripting to parse individual values from this dataset. We excluded data linked with mesenchymal stem cell interventions, focusing solely on the analysis of the monocytes. Quality assurance of the data was confirmed through measures such as a gene expression distribution boxplot for each cohort (Fig. 1A) and a UMAP plot assessing variable distribution across the studied groups (Fig. 1B). Subsequently, we generated a mean-difference plot illustrating the average gene expression levels between normoglycemic controls (control) and type 2 diabetes (T2D) subjects, with the variance between the cohorts represented on the Y-axis (Fig. 1C-D). A subsequent figure was created to spotlight genes that were significantly (*p* < 0.05) and substantially (LogFC > 0.5 or LogFC<-0.5) upregulated or downregulated in the T2D group relative to the control group (Fig. 1E).

For pathway analysis linked with these markedly altered genes in diabetic monocytes, we utilized the online tools available at Metascape.com. Intriguingly, our results indicated that cholesterol metabolism pathways, particularly the Bloch and Kandutsch-Russell pathways, underwent greater modifications in diabetic monocytes/macrophages than pathways responsive to glucose (Fig. 1F). This suggests that cholesterol metabolism may play a pivotal role in dictating macrophage differentiation, polarization, and functionality within a diabetic context.

### Desmosterol elicits a dual phenotypic polarization in macrophages exhibiting both proinflammatory and anti-inflammatory characteristics

**Fig. 2:**
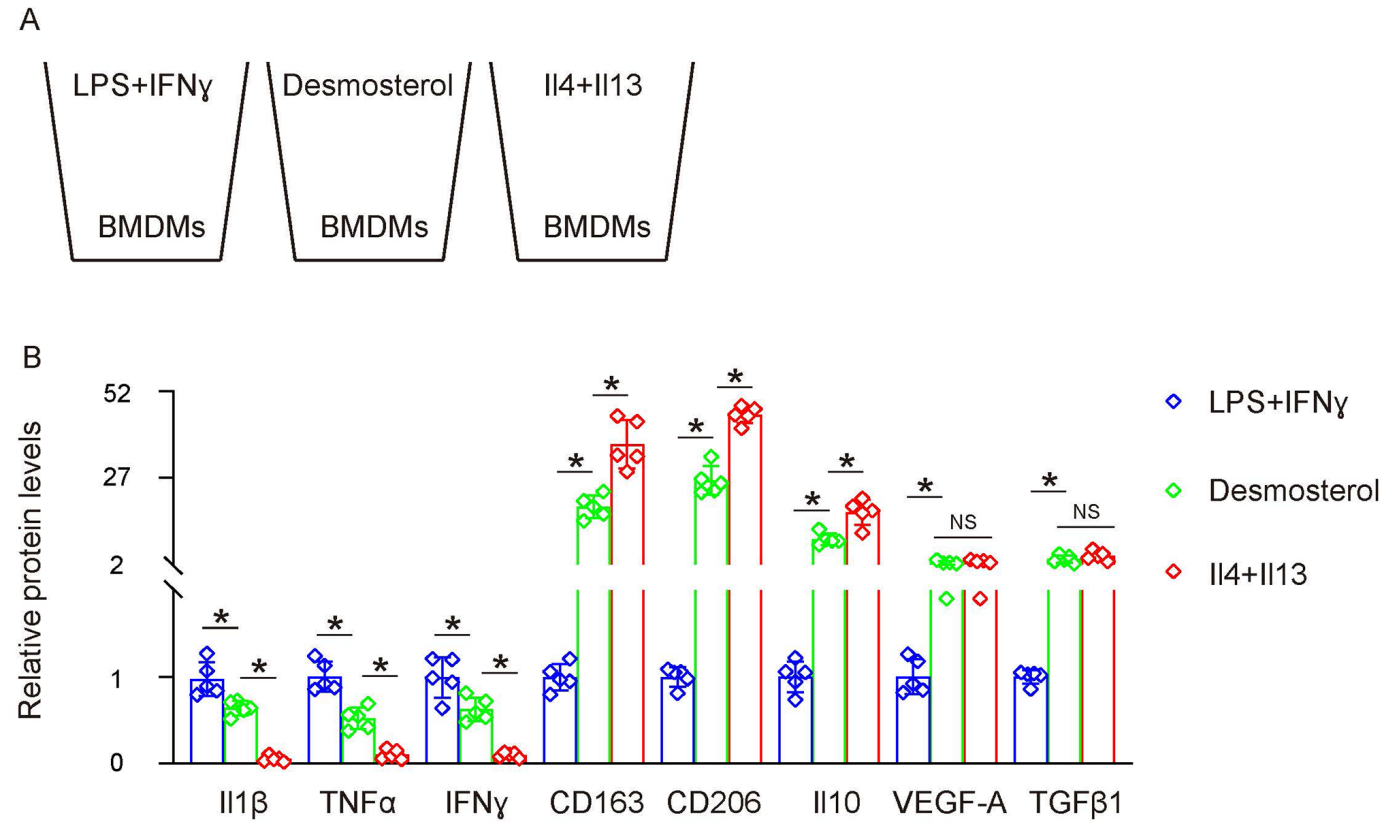
Desmosterol Elicits a Dual Phenotypic Polarization in Macrophages Exhibiting Both Proinflammatory and Anti-Inflammatory Characteristics. **(A)** Mouse bone marrow-derived macrophages (BMDMs) were extracted and exposed to desmosterol. These treated BMDMs were juxtaposed with conventionally polarized M1 (stimulated by LPS and IFN-γ) and M2 (activated by IL-4 and IL-13) macrophages. **(B)** ELISA for key cytokines or factors associated with macrophage polarization in treated macrophages. *N* = 5. *p < 0.05

Desmosterol, an intermediary in cholesterol biosynthesis, has gained attention due to its augmented levels in diabetes and its presumptive role in modulating macrophage activity. Nevertheless, the interplay between cholesterol metabolism, specifically desmosterol’s influence, and macrophage polarization within the realm of DN remains largely uncharted. To elucidate this, we procured mouse bone marrow-derived macrophages (BMDMs) and exposed them to desmosterol. These treated BMDMs were juxtaposed with conventionally polarized M1 (stimulated by LPS and IFN-γ) and M2 (activated by IL-4 and IL-13) macrophages (Fig. 2A). Intriguingly, desmosterol exposure fostered an unconventional polarization of BMDMs. These cells manifested elevated expression levels of pro-inflammatory markers such as Il1β, TNFα, and IFNɣ, hallmarks of M1 macrophages. Concurrently, they also displayed augmented levels of CD163, CD206, Il10, VEGF-A, and TGFβ1, which are distinctive of the anti-inflammatory M2 macrophages (Fig. 2B). These data suggest that desmosterol-driven macrophage polarization yields a hybrid macrophage profile that simultaneously exhibits proinflammatory attributes and potentialities associated with cell proliferation, angiogenesis, and tissue remodeling. This unique macrophage phenotype may have implications for the events observed in diabetes and its associated complications, notably DN.

### Desmosterol-exposed BMDMs simultaneously promote proliferation and apoptosis in podocytes in co-culture

**Fig. 3:**
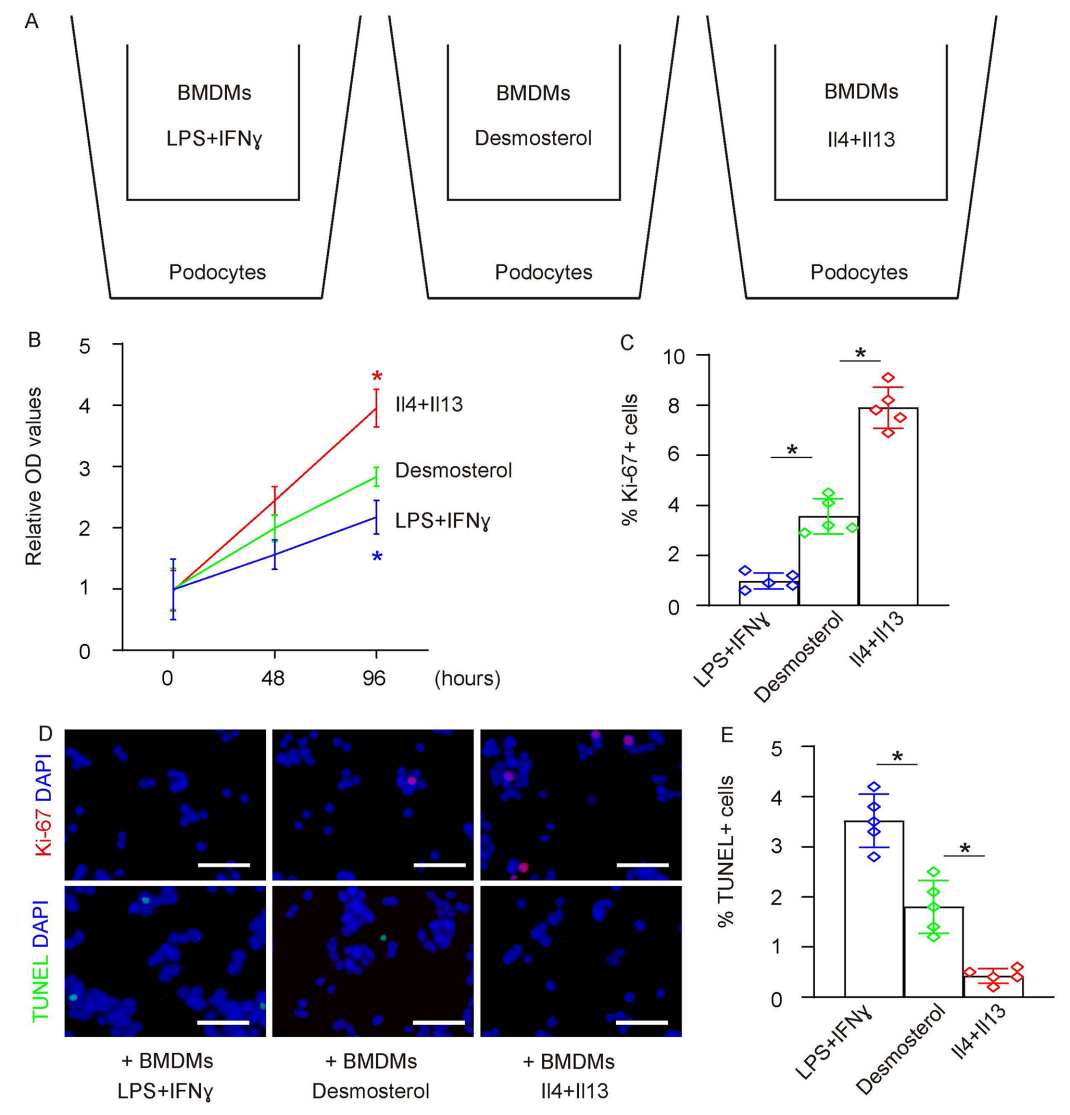
Desmosterol-Exposed BMDMs Simultaneously Promote Proliferation and Apoptosis in Podocytes in co-culture **(A)** Podocytes were co-cultured with desmosterol-exposed BMDMs or traditionally polarized M1 (induced by LPS and IFN-γ) or M2 (stimulated by IL-4 and IL-13) macrophages as controls. **(B)** CCK-8 assay to measure growth of podocytes. **(C)** Ki-67 assay to assess podocyte proliferation, shown by quantification. **(D)** Representative images for Ki-67 and TUNEL staining for podocytes. **(E)** TUNEL assay to assess podocyte apoptosis, shown by quantification. *N* = 5. *p < 0.05. Scale bars were 50 μm

Podocytes are instrumental in the onset of DN. To elucidate the influence of desmosterol-exposed BMDMs on podocytes, we evaluated their effects on the proliferation and apoptosis of co-cultured podocytes. For comparison, traditionally polarized M1 (induced by LPS and IFN-γ) and M2 (stimulated by IL-4 and IL-13) macrophages were used as controls (Fig. 3A). We employed the CCK-8 assay to examine shifts in the total cell count. Podocyte proliferation was determined by measuring the percentage of cells positive for Ki-67, a proliferation indicator, while apoptosis was gauged using the TUNEL assay.

Our findings revealed that podocytes co-cultured with desmosterol-treated BMDMs outnumbered those combined with standard M1 (activated by LPS and IFN-γ) but were fewer than those aligned with M2 (stimulated by IL-4 and IL-13) macrophages (Fig. 3B). Additionally, the proportion of Ki-67 + podocytes co-cultured with desmosterol-treated BMDMs significantly surpassed those with traditional M1 (induced by LPS and IFN-γ), yet notably trailed those coupled with M2 (instigated by IL-4 and IL-13) macrophages (Fig. 3C-D). Concurrently, the fraction of TUNEL + podocytes paired with desmosterol-exposed BMDMs was notably less than with the standard M1 (induced by LPS and IFN-γ), but markedly exceeded those associated with M2 (stimulated by IL-4 and IL-13) macrophages (Fig. 3D-E). These outcomes suggest that desmosterol-treated BMDMs concurrently boost both proliferation and apoptosis in adjoining podocytes, aligning with their cytokine and growth factor expression patterns (Fig. 2B).

### Factors released by desmosterol-treated BMDMs modulate podocyte proliferation and apoptosis in co-culture

**Fig. 4:**
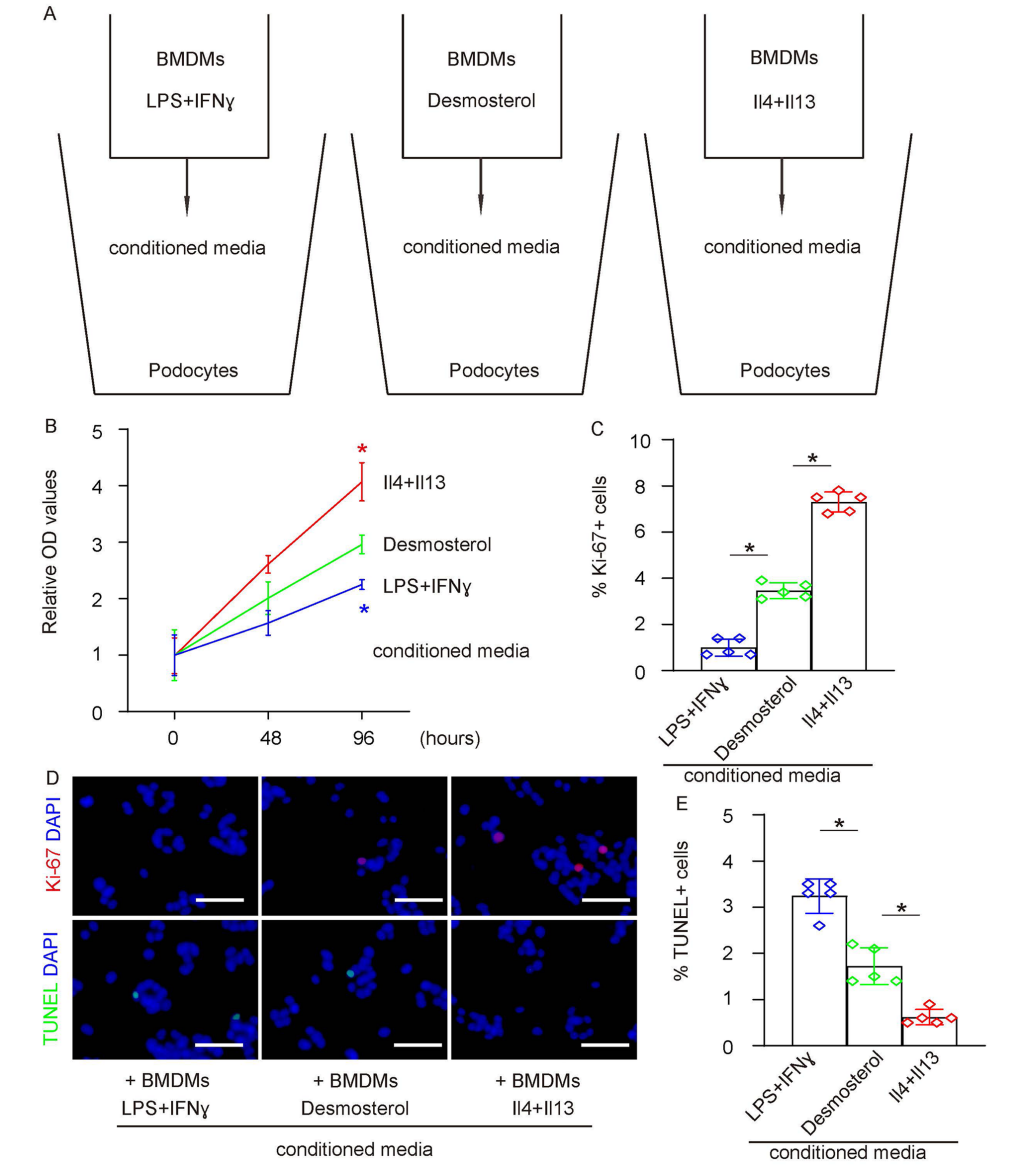
Factors Released by Desmosterol-Treated BMDMs Modulate Podocyte Proliferation and Apoptosis in Co-culture. **(A)** Podocytes were cultured with conditioned media from desmosterol-exposed BMDMs or traditionally polarized M1 (induced by LPS and IFN-γ) or M2 (stimulated by IL-4 and IL-13) macrophages as controls. **(B)** CCK-8 assay to measure growth of podocytes. **(C)** Ki-67 assay to assess podocyte proliferation, shown by quantification. **(D)** Representative images for Ki-67 and TUNEL staining for podocytes. **(E)** TUNEL assay to assess podocyte apoptosis, shown by quantification. *N* = 5. *p < 0.05. Scale bars were 50 μm

We assessed the impact of conditioned media from desmosterol-exposed BMDMs on podocyte behavior (Fig. 4A). Podocytes exposed to this conditioned media exhibited higher counts compared to those subjected to media from conventionally activated M1 macrophages (by LPS and IFN-γ) but were less numerous than podocytes influenced by media from M2 macrophages (activated by IL-4 and IL-13) (Fig. 4B). Notably, the fraction of Ki-67 + proliferating podocytes in the presence of media from desmosterol-treated BMDMs markedly exceeded that with standard M1 media, yet was less than with M2 media (Fig. 4C-D). In parallel, the proportion of TUNEL + apoptotic podocytes was significantly reduced when co-cultured with media from desmosterol-exposed BMDMs, compared to standard M1 media. However, this fraction was considerably higher than that observed with M2 media (Fig. 4D-E). These results intimate that the modulatory effects of desmosterol-exposed BMDMs on podocytes arise from the release of specific cytokines and growth factors.

**Fig. 5:**
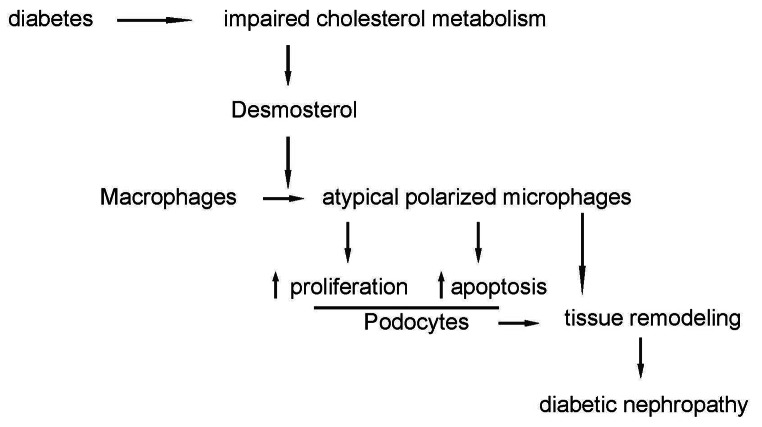
Schematic of the study. Disrupted cholesterol metabolism in diabetes can trigger an unconventional macrophage polarization via cholesterol intermediates like desmosterol. This polarization potentially influences podocyte proliferation and apoptosis, reshaping renal tissue and contributing to the onset of DN.

In conclusion, our research posits that disrupted cholesterol metabolism in diabetes can trigger an unconventional macrophage polarization via cholesterol intermediates like desmosterol. This polarization potentially influences podocyte proliferation and apoptosis, reshaping renal tissue and contributing to the onset of DN (Fig. 5).

## Discussion

DN remains a chief complication of diabetes, representing a pivotal cause of end-stage renal disease worldwide [2]. Among the cellular actors in this pathology, podocytes, a type of specialized epithelial cell, have emerged as critical contributors to the progression of DN [8–12]. Podocytes, with their intricate foot processes, maintain the integrity of the glomerular filtration barrier, a function indispensable for kidney homeostasis [19]. In DN, these cells undergo significant morphological and functional transformations, such as foot process effacement, detachment from the glomerular basement membrane, and apoptosis [19]. These alterations compromise the filtration barrier, leading to albuminuria, a hallmark of DN [19]. Furthermore, podocyte loss due to apoptosis directly correlates with the progression of glomerulosclerosis, emphasizing their pivotal role in DN pathogenesis [19].

Macrophages play diverse roles in various renal pathologies, including DN. In the diabetic milieu, macrophage infiltration into the kidney, particularly the glomeruli, has been widely observed [20]. These infiltrating macrophages, depending on their polarization state, can either exacerbate inflammation and injury or promote repair and resolution [3]. For instance, M1 macrophages, characterized by their pro-inflammatory properties, can release cytokines and chemokines that could potentially harm podocytes, aggravate proteinuria, and advance glomerular sclerosis. On the other hand, M2 macrophages, which possess anti-inflammatory and reparative qualities, might promote tissue repair [3]. However, the novel observation of an ‘atypical’ macrophage polarization induced by desmosterol suggests that macrophages can adopt a spectrum of phenotypes, and their precise role in influencing podocyte behavior might be more nuanced than the conventional M1/M2 dichotomy [21].

Diabetes, characterized by chronic hyperglycemia, significantly disrupts cholesterol metabolism, leading to various metabolic impairments [22]. In diabetes, insulin resistance or deficiency alters lipid metabolism, often resulting in dyslipidemia [22]. This condition is marked by elevated levels of LDL (low-density lipoprotein) cholesterol and triglycerides, alongside reduced HDL (high-density lipoprotein) cholesterol [22]. Insulin resistance hampers the normal function of lipoprotein lipase, an enzyme crucial for triglyceride breakdown, leading to their accumulation [22]. Additionally, the hepatic synthesis of cholesterol is often increased in diabetic conditions due to insulin’s diminished inhibitory effect on key enzymes like HMG-CoA reductase [22]. Furthermore, chronic inflammation and oxidative stress, common in diabetes, exacerbate the dysfunction in cholesterol metabolism, contributing to the formation of oxidized LDL, a risk factor for cardiovascular complications [22]. This disrupted cholesterol homeostasis in diabetes underscores the interplay between metabolic diseases and lipid metabolism.

The mechanism by which desmosterol influences macrophage polarization remains to be fully elucidated. Considering its role as a cholesterol synthesis intermediate, desmosterol could impact cellular membranes’ fluidity and lipid raft formation, subsequently influencing various signaling pathways [23]. It could modulate the activity of certain transcription factors, possibly via the LXR (Liver X Receptor) pathway, which is known to be influenced by oxysterols and has been implicated in macrophage function [24]. Moreover, other signaling molecules like PPARγ [25] and NF-κB [26], which play roles in macrophage polarization and function, might also be affected. The precise integration of these pathways in response to desmosterol is a new area for future investigation. For example, microRNAs (miRNAs) are small, non-coding RNA molecules that play a critical role in gene regulation and are key players in the pathophysiology of various diseases, including DN [27]. Adapted macrophages can release miRNAs either directly as soluble factors in association with cytokines or packaged within exosomes [27]. These exosomes, as vehicles for miRNA transport, ensure stability and facilitate targeted delivery to podocytes and endothelial cells [27]. Once inside these cells, miRNAs can regulate gene expression post-transcriptionally, either inhibiting mRNA translation or leading to mRNA degradation, to modulate key processes such as inflammation, fibrosis, and cellular apoptosis [27].

Here, we showed that the desmosterol-driven alteration in macrophage polarization affects podocyte dynamics in DN. On the other hand, the podocytes may also regulate macrophage polarization through various signaling mechanisms. They secrete cytokines and chemokines, such as IL-6 and Chemokine (C-C motif) ligand 2 (CCL2), which can recruit macrophages and often promote a pro-inflammatory M1 phenotype, particularly in disease states like DN [28]. Additionally, podocytes produce growth factors like TGFβ1, known to drive macrophages towards an M2, tissue-repairing phenotype. Moreover, the release of extracellular vesicles from podocytes, including exosomes containing microRNAs and proteins, also plays a role in modulating macrophage activity [29]. Interactions through cell surface receptors and ligands, along with the release of metabolic by-products in conditions of stress, further contribute to the regulatory influence of podocytes on macrophage polarization [29].

As with all scientific research, our study has inherent limitations. Firstly, while the data underscores an association between desmosterol and macrophage polarization, a direct causative relationship needs to be established. Our in vitro findings, though compelling, might not fully recapitulate the complex in vivo microenvironment of the diabetic kidney. The use of conditioned media, while providing valuable insights, lacks the intricate cell-cell interactions that could influence macrophage-podocyte interplay in an actual diabetic setting. Moreover, our reliance on public databases for initial gene expression analyses, although beneficial, is predicated on the assumption that the patient cohorts are representative and that the methodologies employed were rigorous.

Despite the limitations, our findings provide a promising avenue for therapeutic interventions in DN. The revelation that cholesterol metabolism, and not just glucose pathways, is significantly altered in diabetic macrophages could pave the way for novel therapeutic targets. If desmosterol-induced macrophage polarization is indeed proven to be causative in DN progression, modulating this pathway might ameliorate disease outcomes. This could be achieved using small molecules, antibodies, or even leveraging advancements in gene therapy [30]. Ultimately, understanding the intricate dance between podocytes and macrophages in the diabetic milieu could be the key to unlocking innovative treatments for DN.

## Conclusions

In conclusion, our study provides fresh insights into the multifaceted interplay between podocytes, macrophages, and cholesterol metabolism in DN. While several questions remain, the findings present intriguing prospects for therapeutic advancements in combating this debilitating renal complication of diabetes.

## Data Availability

The datasets generated during and/or analyzed during the current study are available from the corresponding author on reasonable request.
